# Fabrication of Chiral 3D Microstructure Using Tightly Focused Multiramp Helico-Conical Optical Beams

**DOI:** 10.3390/mi13101771

**Published:** 2022-10-18

**Authors:** Jisen Wen, Qiuyuan Sun, Mengdi Luo, Chengpeng Ma, Zhenyao Yang, Chenyi Su, Chun Cao, Dazhao Zhu, Chenliang Ding, Liang Xu, Cuifang Kuang, Xu Liu

**Affiliations:** 1 Research Center for Intelligent Chips and Devices, Zhejiang Lab, Hangzhou 311121, China; 2 State Key Laboratory of Modern Optical Instrumentations, Zhejiang University, Hangzhou 310027, China

**Keywords:** direct laser writing, two-photon polymerization, multiramp helical–conical beam, chiral microstructure

## Abstract

Beams with optical vortices are widely used in various fields, including optical communication, optical manipulation and trapping, and, especially in recent years, in the processing of nanoscale structures. However, circular vortex beams are difficult to use for the processing of chiral micro and nanostructures. This paper introduces a multiramp helical–conical beam that can produce a three-dimensional spiral light field in a tightly focused system. Using this spiral light beam and the two-photon direct writing technique, micro–nano structures with chiral characteristics in space can be directly written under a single exposure. The fabrication efficiency is more than 20 times higher than the conventional point-by-point writing strategy. The tightly focused properties of the light field were utilized to analyze the field-dependent properties of the micro–nano structure, such as the number of multiramp mixed screw-edge dislocations. Our results enrich the means of two-photon polymerization technology and provide a simple and stable way for the micromachining of chiral microstructures, which may have a wide range of applications in optical tweezers, optical communications, and metasurfaces.

## 1. Introduction

Interest in three-dimensional (3D) micromachining is driven by many advanced applications in different fields, such as materials science [1,2,3,4], biology [5], and photonics [6,7,8]. Many research groups have made great efforts in developing and improving direct writing techniques that can produce 3D objects with a high spatial fidelity and resolution. Examples of these advances can be found in many fabrication techniques using energy beams (for example, photons [8] and electrons [9,10]) and ink streams [11]. Femtosecond laser microfabrication using two-photon polymerization (TPP) is the most particularly attractive technique for fabricating micro and nanostructured components and functional microdevices because of its 3D processing capability and high spatial resolution. Three-dimensional micro and nanostructures with a small size and high level of integration have a variety of applications in photonic crystals [6,7,12], cell biology [13], metamaterials [14,15], microcavities [16], and microfluidics [17]. Maruo et al. pioneered the study on 3D microfabrication with TPP via a pulsed beam with a 200 fs pulse duration time [8]. In 2002, Straub et al. used the TPP to print near-infrared photonic crystals, which showed evidence of photonic bandgaps in photopolymer microstructures in the near-infrared region [18].

While this technique can produce very-high-resolution 3D structures, its main disadvantage is its inefficiency due to the serial nature of point-by-point processing. Thus, two strategies were proposed to break through the limitation of the low efficiency of point-by-point processing. One of the methods that researchers have proposed is parallel micro–nano lithography technology with a multifocal optical beam. For example, multi-beam interference is used for specific periodic structures [19,20]. Gittard et al. verified that multiple beams simultaneously produce multiple microstructures, effectively reducing the fabrication time of 3D medical devices [21,22]. Li et al. used an array of hollow spots, including four doughnut-shaped spots generated by a spatial light modulator (SLM), to realize parallel STED-based TPP for big data recording [23]. Recently, Geng et al. utilized an ultrafast random-access digital micromirror device scanner for parallel nanofabrication [24]. Maibohm et al. demonstrated parallelization TPP through a fixed DOE cutting the fabrication time to a fraction for large unit structures [25].

Another strategy is using 2/3D-structured light field projection to further increase the writing speed, especially when processing special 3D structures or functional devices [26]. Zhang et al. made use of a ring-shaped light field to print a tube structure array for a single exposure that has a higher fabrication efficiency than parallel fabrication using an array of beam spots [27]. Liang et al. applied the Bessel beams for fabricating the uniform cylinder microstructures [28]. Flower-like microstructures printed by superposed Bessel beams were also reported [29]. On the other hand, some spiral and chiral microstructures that have potential applications in optoelectronic devices and analytical chemistry were made by a single exposure of the structured light field. Zhang et al. polymerized complex 3D structures using the focus of a vortex beam carrying orbital angular momentum [30]. Exploiting the coaxial interference of a vortex beam and a plane wave, Ni et al. fabricated chiral microstructures using structured optical vortices, showing a spiral intensity in 3D space [31]. Xin et al. fabricated hollow microhelices based on a femtosecond vortex beam that has flexible control regarding heights, diameters, pitch numbers, taper angles, and pitch periods [32]. Yang et al. proposed a novel method for designing a kind of magnetic 3D tubular micromotor using a single exposure of structured optical vortices in a magnetic photoresist that has a sub-micrometer resolution as well as a high uniformity, but disadvantages in the costs and requirements of specific equipment [33]. Pan et al. demonstrated the rapid fabrication of 3D chiral microstructures via a chiral discrete vortex beam generated by the interference of multiple parallel vortex beams. This work greatly improves the fabrication of such a structure efficiency by approximately 100 times [34]. Cheng et al. adopted structured beams with the shapes superposition of high-order Bessel modes to pattern helical structures with a tunable axial that improves the mass microfabrication over large surface areas [35].

The helical–conical beams (HCBs), a kind of structured light field, have a spiral intensity at the focal plane resulting from a special phase that is a product of a helical and a conical phase. It was first studied on its propagation of the HCBs in the focusing system in 2005 by Alonzo et al. [36]. Hermosa et al. mapped out complicated phase structures of HCBs produced via computer-generated holograms [37]. Singh et al. further compared the vortices in the phase of the HCBs with the fractional vortex beam [38]. Nestor et al. found that vortices of HCBs have significant induced angular rates of motion by tracking the vortices at several distances using scalar diffraction theory [39]. By means of the characteristic of the spiral profile on the amplitude of HCBs, Daria et al. successfully trapped microparticles to make the spiral motion [40]. Then, the self-healing properties of the HCBs that come from the transverse helical energy flow were also observed experimentally [41,42]. Recently, cosh-Gaussian HCBs, power-exponent HCBs, and bored HCBs have been proposed [43,44,45]. The generation of HCBs by a plasmonic metasurface was also demonstrated [46]. Although there are some works related to the evolution properties of the HCBs as introduced above, there are few studies on the propagation properties in a high-NA system. Moreover, the application of the HCBs in the fabrication of 3D structures by TPP has not yet been realized.

In this paper, we introduce a new kind of HCBs: so-called multiramp helical conical beams (MHCBs). The MHCBs passing through a high numerical aperture objective lens were studied both numerically and experimentally. It was found that MHCBs with multiramp phases have a 3D spiral intensity distribution. The unique characteristics of this beam focus were exploited to perform single exposures for the successful micromachining of chiral microstructures through a two-photon polymerization effect. To the best of our knowledge, it is the first time an MHCB is adopted to realize the 3D chiral micro-structure. We believe that it is useful in direct laser writing for fabricating 3D chiral structures.

## 2. Theory

**Figure 1:**
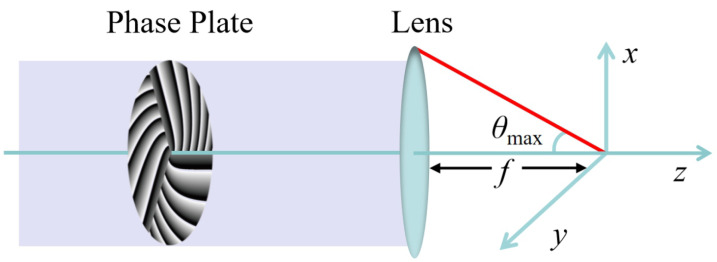
Schematic diagram of tight focusing of MHCBs.

In order to fabricate the chiral microstructure using MHCBs, let us first start to study the propagation properties of MHCBs in a high-NA lens-focusing system as shown in Figure 1. Figure 1 illustrates the geometry of a tightly focused system. The incident light beam goes through the designed phase plate and is focused by a high-NA lens with focal length *f*. The height of the incident light beam is *h*. Thus, h=fsinθ, where θ is the angle between the ray direction and the optical axis. Furthermore, $0<\theta <\theta_{\max}$, where $\theta_{\max}=\arcsin\left(\mathrm{NA}/n\right)$. Here, we consider a plane wave or fundamental Gaussian beam as an incident beam and let it reflect from a diffractive phase element; for example, a phase plate or phase-modulated spatial light modulator (SLM). Then, the light field at the initial plane can be simply given as

(1) $$E_{\mathrm{in}}\left(r,\phi \right)=A_{0}\left(r,\phi \right)t\left(\phi \right),$$

where (r,ϕ,z) are the cylindrical coordinates at the input plane and $A_{0}=\exp\left(-\frac{r^{2}}{w_{0}^{2}}\right)$, or 1 for a plane wave or fundamental Gaussian beam, respectively, where $w_{0}$ is a half-width beam. t(ϕ) is the transmission function of the phase. For MCHBs, the multiramp helical–conical phase is assumed to be

(2) $$t\left(\phi \right)=\exp\left[i\alpha \left(\frac{r}{r_{0}}-K\right)\left(\phi -\frac{2\pi n}{m}\right)\right],$$

where *K* is a constant that takes a value of either 1 or 0, $r_{0}$ is a normalization factor of the radial coordinate, α is the winding number around the azimuth angle, *m* is an integer representing the number of multiramp mixed screw-edge dislocations, and $n=\left\lfloor m\phi /(2\pi )\right\rfloor$ with $\left\lfloor \cdot \right\rfloor$ is the floor function, respectively. It can be seen from Equation (2) that the phase function has a nonseparable radial and azimuthal phase dependence. In the next discussion, we will only focus on the MHCBs in the case of K=0.

**Figure 2:**
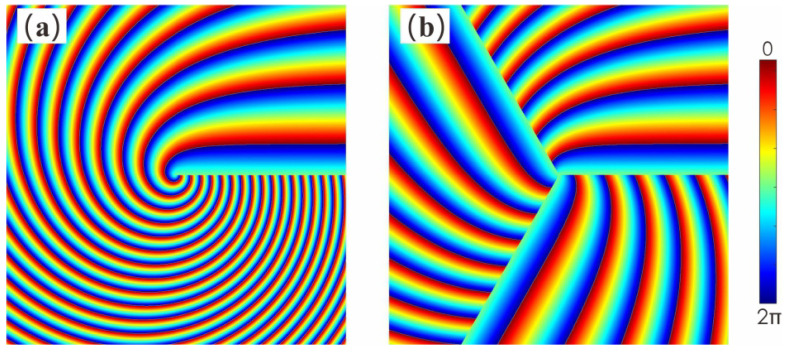
Phase distributions of (**a**) MHCBs with K=0,m=1,α=6 and (**b**) MHCBs with K=0,m=3,α=6.

According to Equation (1), the phase distribution of MHCBs is plotted. The phase described by Equation (1), while similar to the phase of the fractional vortex beam, is totally different because the phase step varies linearly with *r* and the phase becomes continuous around an azimuthal circuit at $r_{0}$. Figure 2 shows the comparison of phase distributions of the two MHCBs. Figure 2a shows the phase distribution of the MHCBs with the number of multiramp m=1 and α=6, which is a traditional HCB, whereas Figure 2b shows the phase distribution of the MHCBs with m=3 and α=6. Moreover, the phase distribution of MHCBs with α=6 is divided into three parts. Each part of the phase can be used to generate HCBs.

Based on the vectorial diffraction theory [47], the tightly focused field of the incident beam with different amplitude, phase, and polarization states can be expressed as

(3) $$\begin{array}{cc} E_{\mathrm{out}}\left(\rho ,\varphi ,z\right) & =\left[\begin{array}{c} E_{x}\\ E_{y}\\ E_{z} \end{array}\right]=iC\int \int_{\Omega }\sin\left(\theta \right)\cdot E_{\mathrm{in}}\left(r,\phi \right)\cdot A\left(\theta ,\phi \right)\cdot \left[\begin{array}{c} p_{x}\\ p_{y}\\ p_{z} \end{array}\right]\\ \; & \times e^{ikn\left[z\cos\theta +\rho \sin\theta \cos(\phi -\varphi )\right]}d\theta d\phi , \end{array}$$

where $\left(\rho ,\varphi ,z\right)$ are the coordinates at the output plane, and $E_{\mathrm{out}}\left(\rho ,\varphi ,z\right)$ is the output light field near the focal plane of a high-NA lens. ${\left[E_{x}\;E_{y}\;E_{z}\right]}^{T}$ is the output light field represented in the Cartesian coordinate system. *C* is the normalized constant. θ is the angle between the ray direction and the optical axis. Furthermore, $0\leq \theta \leq \theta_{\max}$, where $\theta_{\max}=\arcsin($NA/n) and the integral upper limit $\alpha \;=\theta_{\max}$, where NA is the numerical aperture and *n* is the refractive index of the focal region. *k* is the wave number of the focal region. ${\left[p_{x}\;p_{y}\;p_{z}\right]}^{T}$ is a unit vector about the polarization of the incident MHCBs. Furthermore, A(θ,ϕ)=a(θ)V(θ,ϕ), and it is a 3×3 matrix, where

(4) $$a\left(\theta \right)=\sqrt{\cos\theta },$$

(5) $$V\left(\theta ,\phi \right)=\left[\begin{array}{ccc} 1+\left(\cos\theta -1\right)\cos^{2}\phi & (\cos\theta -1)\cos\phi \sin\phi & -\sin\phi \cos\theta \\ (\cos\theta -1)\cos\phi \sin\phi & 1+\left(\cos\theta -1\right)\sin^{2}\phi & -\sin\phi \cos\theta \\ \sin\theta \cos\phi & \sin\theta \cos\phi & \cos\theta \end{array}\right].$$

Then, the total intensity of the tightly focused MHCBs can be expressed as

(6) $$\begin{array}{cc} I_{out} & =I_{x}+I_{y}+I_{z}\\ \; & =|E_{x}{|}^{2}+|E_{y}{|}^{2}+{\left|E_{z}\right|}^{2}. \end{array}$$

## 3. Numerical Simulations and Experimental Results

**Figure 3:**
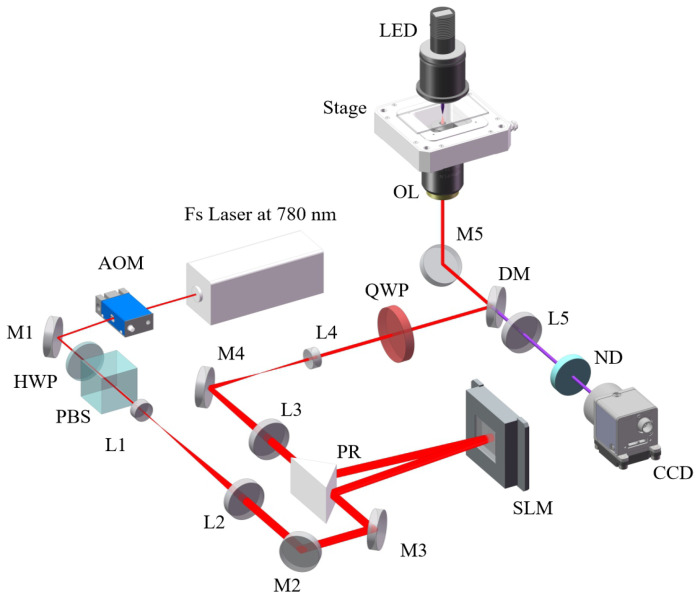
Experimental setup for laser direct writing by MHCBs. Other notations are: HWP, half-wave plate; PBS, polarized beam splitter; M, mirror; SLM, spatial light modulator; QWP, quarter-wave plate; L, lens; AOM, acoustic–optical modulator; PR, prism reflector; OL, objective lens; DM, dichromatic mirror; ND, neutral density filter.

We used this kind of beam to fabricate the micro-structure based on TPP. The laser direct writing system in our experiments is schematically shown in Figure 3. Experimentally, the femtosecond laser source is a Ti: sapphire laser with dispersion precompensation (Chameleon Vision II, from Coherent Inc., Santa Clara, CA, USA), with a central wavelength of 780 nm, a pulse width of 140 fs, and a repetition rate of 80 MHz. MHCBs are generated by a reflection-type phase-modulation SLM (SLM, X15223-02 LCOS-SLM, Hamamatsu, Pohotonics, K.K., Hamamatsu, Japan) with 1276 × 1024 pixels, 12.5 μm pixel pitch and have 15.9 mm × 12.8 mm of the active area in the experiment. The linearly polarized beam from a femtosecond (with λ=780 nm) first passes through AOM (MT110-A1.5-IR, AAOpto-Electronic, Orsay, France) worked at 110 MHz frequency, and the first-order diffracted beam is selected. AOM works as a high-speed optical switch to turn on/off the incident light beam. A half-wave plate and a polarized beam splitter were used to control the intensity and polarization of light. A beam expander (L1, L2) was used to expand the optical beam width to 2 mm. The expanded beam was reflected by a prism reflector onto the face of SLM, with the incident angle at 5 degrees. Here, the phase pattern on the SLM needs to be corrected by a background-compensated phase due to the imperfect flatness of the SLM surface and the wavefront of the incident beam. The SLM was placed at the front focal plane of L3. L3 and L4 form a 4f system to image the modulated light MHCBs into the front focal plane of the objective lens (OL, 100×/1.45 NA, Nikon, Tokyo, Japan). The quarter wave plate converts linear polarization to circular polarization. Finally, the MHCBs were focused onto a sample located under the focal plane of an objective lens, and a chiral microstructure was achieved under single exposure. The modulated MHCBs and the fabricated sample can be viewed by a CCD camera. The neutral density filter was used to protect the CCD from damage by the high optical intensity. The sample was mounted on a 3D adjustable platform (E545, from Physik Instrumente (PI) GmbH and Co. KG, Karlsruhe, Germany) with a 2 nm resolution and a 300 μm × 300 μm × 300 μm moving range to precisely locate microstructures. After fabricating, the samples were developed in polyethylene glycol methyl ether acetate (8 min), isopropyl alcohol (2 min).

**Figure 4:**
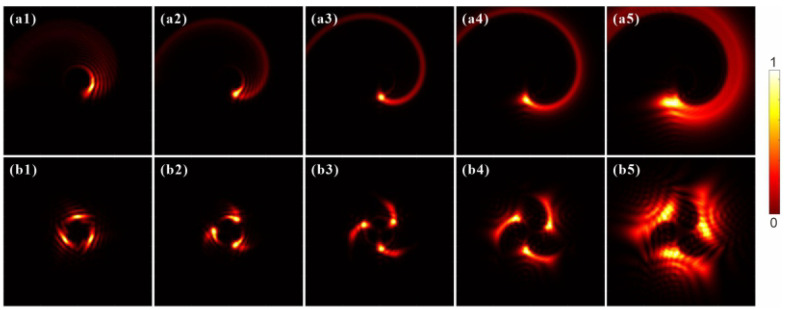
Simulated light field of MHCBs at the focal region of high numerical aperture (NA = 1.45) oil-immersion objective lens. From top to bottom, the tightly focused MHCBs with parameter (**a1**–**a5**) m=1,α=6 and (**b1**–**b5**) m=3,α=6. Simulated cross-sectional intensity profiles for the MHCBs at different positions through the objective lens (**a1**,**b1**) z=−2λ, (**a2**,**b2**) z=−λ, (**a3**,**b3**) z=0, (**a4**,**b4**) z=λ, (**a5**,**b5**) z=2λ.

There are some previous works related to the evolutions of the MHCBs. Strictly speaking, those beams with helical–conical phases are different from our design since we used the phase function given by Equation (2), which possesses a multiramp structure. Moreover, no work has been carried out experimentally on investigating the tight focusing property of such a kind of beam. Therefore, in order to understand the propagation properties of the MHCBs in the tightly focused system, we have simulated intensity distributions near the focal plane of the objective lens in our direct laser writing system. Figure 4 plots the numerical intensity distributions of the tightly focused MHCBs when they gradually approach and move away from the focal plane of the objective lens, which is shown in Figure 3. The focusing was carried out with a high-NA oil-immersion objective lens and the refractive index of the oil was n=1.518. As the beam propagates in such a high-NA system, its intensity profile changes with respect to the propagation distance rapidly. It is observed in Figure 4a1 that, when MHCB with parameter K=0,m=3,α=6 is at a distance of z=−2λ away from the focal plane, there exists a curved moon-shaped main lobe of light intensity rotating along the center of the optical axis, and some weaker side lobe light intensity distribution is in the periphery. As the beam approaches the focal plane—see Figure 4a1,a2—the strongest light intensity in the main lobe of light intensity rotates around the optical axis. At the same time, the peripheral side lobes contract together. The intensity distribution at the focal plane is very clearly observed as a spiral shape as shown in Figure 4a3. As the light passes through the focal plane, the spiral light intensity gradually spreads out to form a wider band-like light intensity distribution, and, at the same time, the center of the strongest light intensity rotates around the optical axis in Figure 4a4,a5. For the MHCB with m=3,α=6, three main lobes with some side lobes can be observed in the intensity profile in Figure 4b1,b2 when z=−2λ. As it approaches the focal plane, three spiral shape intensity distributions are clearly seen; see Figure 4b3. The location of the maximum intensity in the three main lobes continuously twists around the central propagation optical axis, which is similar to the MHCB with m=1,α=6. As it propagates to the position at z=2λ, the side lobes near the main lobe appear again and there are some interference strips between the three main lobes. It is also the difference between MHCBs with m=3,α=6 and m=1,α=6. Furthermore, it can be concluded that the number of main lobes is dependent on the number of multiramp mixed screw-edge dislocations *m*.

**Figure 5:**
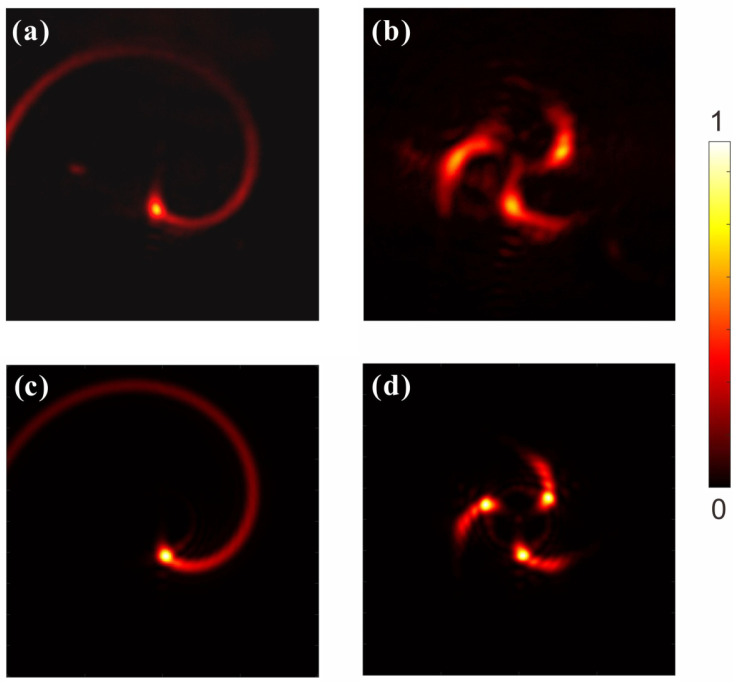
Measured and numerical calculated intensity profiles of MHCBs with parameter (**a**,**c**) m=1,α=6 and (**b**,**d**) m=3,α=6 at the focal plane of the objective lens.

Figure 5 shows the experimental and numerical simulation results of the MHCBs propagating in the high-NA objective lens system for direct laser writing. Figure 5 shows the comparison of the actual images taken on the CCD camera with the numerical results for MHCBs under different parameters. Figure 5a,c show the experimental and theoretical intensity distributions, respectively, for the MHCB with m=1 and α=6 at the focal plane z=0 of the objective lens. It is clearly seen that the measured intensity profile is in good agreement with the theoretical result. The intensity distribution on the focal plane is very clearly represented as a spiral shape. Similar results can also be found in the case of MHCBs with m=3 and α=6.

**Figure 6:**
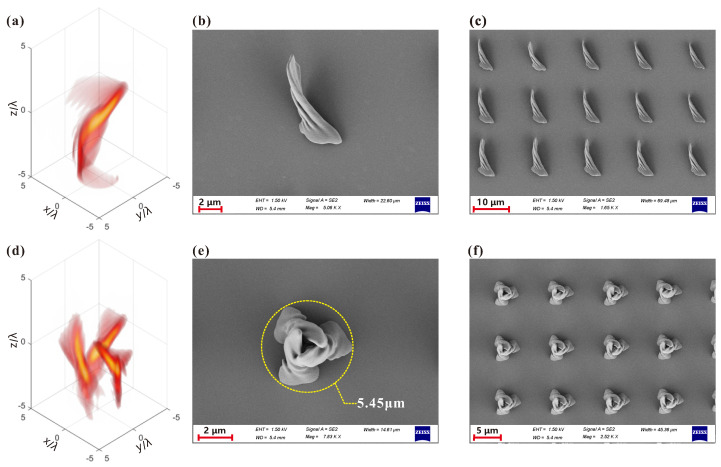
The 3D structure of light field of the MHCBs and corresponding fabricated chiral structure. The 3D intensity distribution of the MHCBs with (**a**) m=1,α=6 and (**d**) m=3,α=6. SEM photos of the fabricated 3D chiral microstructures via tightly focused MHCBs with (**b**) m=1,α=6 and (**e**) m=3,α=6. SEM photos of the fabricated 3D chiral microstructures array via MHCBs with (**c**) m=1,α=6 and (**f**) m=3,α=6.

After understanding the tightly focused properties of MHCBs in an objective lens system, the MHCBs wre utilized to fabricate 3D chiral structure in the direct-laser-writing-system-based TPP as shown in Figure 3. The left column of Figure 6 shows the 3D intensity distribution of MHCBs with different parameters, which are used to fabricate the chiral microstructures. The resulting chiral structures were imaged by scanning electron microscopy (SEM) as shown in the middle and right columns of Figure 6 under the single exposure time t=250 ms and used laser power P=35.96 mW. It is seen that the chiral microstructure with only one subunit was fabricated when using MHCB with m=1,α=6. However, when MHCB with m=3,α=6, the chiral microstructure tat consists of three subunits was fabricated. In other words, the number of the subunits of the chiral microstructure is dependent on the parameter *m* on MHCBs. The outer diameter of such a chiral microstructure is approximately 5.45 μm, which is marked by a dashed circle as shown in Figure 6e. In terms of the fabrication volume per unit time, our method is more than 20 times higher than point-by-point scanning for similar structures (detailed discussion in Appendix A). By moving the 3D adjustable platform collaboration with AOM as an optical switch, repeated chiral microstructures were fabricated, as shown in Figure 6c,f, which forms an array. These chiral microstructure arrays in possession of chirality may have great applications in optics sensing and optics detection [48].

**Figure 7:**
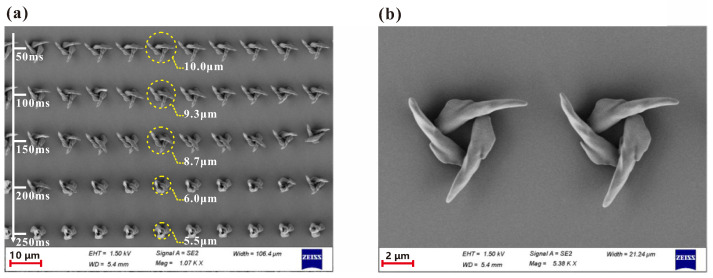
SEM photos of the fabricated 3D chiral microstructures under different exposure times. (**a**) The exposure time increases with a step of 50 ms along the white arrow. (**b**) Two Chiral microstructures were fabricated under an exposure time of 50 ms.

Figure 7 shows the effect of the single exposure time on the fabrication results of the chiral microstructure using MHCBs. We increased the single exposure time from 50 ms to 250 ms in increments of 50 ms as the white arrow shows in Figure 7a, whereas the used laser power was kept constant at P=35.96. It was observed that the outer diameter decreases with the increase in the exposure time. However, the relationship between the outer diameter of the structure and exposure time is not linear. For example, when the exposure time is 50 ms, the outer diameter of the microstructure is approximately 10 μm, whereas the outer diameter of the microstructure reaches 5.5 μm with an exposure time of 250 ms. It can be explained that, when the higher exposure time is used, subunits of chiral microstructure tip towards the center and form stable microstructures. However, subunits tip towards the periphery when using a lower exposure time as shown clearly in Figure 7b.

## 4. Conclusions

We have demonstrated a rapid fabrication method for 3D chiral microstructures with tightly focused MHCBs base on TPP for the first time. The multiramp helical–conical phase was proposed to generate MHCBs. Tightly focused MHCBs in an objective lens system were theoretically analyzed by the vectorial diffraction theory. They were generated experimentally by using a multiramp helical phase loaded on an SLM, which had a good agreement with the theoretical result. Three-dimensional spiral optical fields were produced by passing through a high-NA focusing system, and 3D chiral microstructures with controllable spiral lobes were polymerized after the illumination of MHCBs. The effect of the exposure time on the outer diameter of the fabricated chiral structure was studied and it was found that the outer diameter decreases with the increase in the exposure time. Compared to conventional point-by-point scanning fabrication, the reported result of the fabrication efficiency is more than 20 times higher for fabricating similar structures. Although our method may have a shortage in making high chiral structures, and poor flexibility, it provides a new promising technique to fabricate chiral microstructures that may have potential applications in metamaterials, microfluidics, microrobots, and biomaterials. Moreover, the method proposed is a step forward for mass microfabrication over large surface areas.

## Figures and Tables

**Figure A1:**
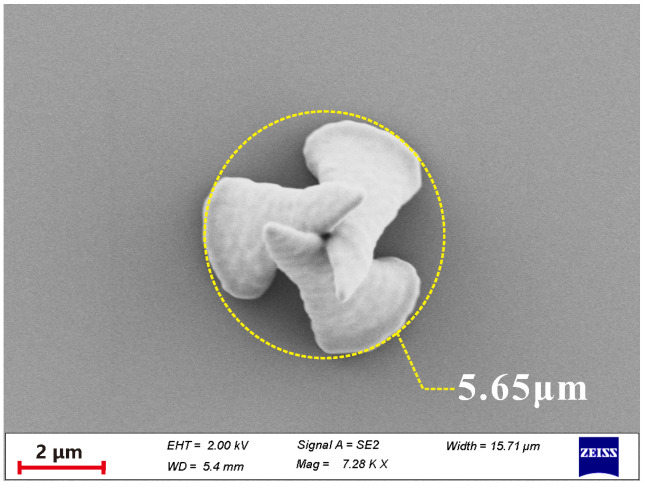
SEM photo of 3D chiral microstructure fabricated by conventional point-by-point scanning TPP.

## Data Availability

Not applicable.

## Appendix A. Chiral Microstructure Fabricated by Conventional Point-by-Point Scanning TPP

A similar chiral microstructure to the fabricated structure shown in Figure 7 was fabricated by conventional point-by-point TPP. The precision of the 3D graphic slice was 100 nm, and the scanning speed of the galvanometer was 20 m/s, which is the highest scanning speed of our self-developed TPP system. Therefore, the total fabrication time was 5000 ms.
